# Differentiation between Solitary Cerebral Metastasis and Astrocytoma on the Basis of Subventricular Zone Involvement on Magnetic Resonance Imaging

**DOI:** 10.1371/journal.pone.0133480

**Published:** 2015-07-21

**Authors:** Rong Wang, Jiaqi Ma, Gang Niu, Jie Zheng, Zhe Liu, Yonghao Du, Bolang Yu, Jian Yang

**Affiliations:** 1 Radiology Department of the First Affiliated Hospital, Xi’an Jiaotong University, Xi’an, Shaanxi, People’s Republic of China; 2 Clinical research center of the First Affiliated Hospital, Xi’an Jiaotong University, Xi’an, Shaanxi, People’s Republic of China; National Yang-Ming University, TAIWAN

## Abstract

**Purpose:**

To determine the relationship between the subventricular zone (SVZ) and astrocytoma based on magnetic resonance imaging (MRI) and whether SVZ involvement can be used to distinguish solitary cerebral metastases (SCMs) from astrocytomas.

**Methods:**

This retrospective study involved 154 patients with solitary low-grade astrocytoma (LGA), high-grade astrocytoma (HGA), and SCM, who underwent T1-weighted imaging (T1WI), Gd-DTPA–enhanced T1WI, and T2-weighted imaging (T2WI) or fluid-attenuated inversion recovery (FLAIR) T2WI. The spatial relationship between the tumor and SVZ was classified as “involvement” or “segregation” on contrast-enhanced T1WI for enhanced tumors and T2WI/FLAIR T2WI for non-enhanced tumors. Patient-based SVZ-contact rates were compared between the LGA, HGA, and SCM groups. The frequencies of involvement of various lateral ventricle regions by astrocytoma were compared. The correlation between SVZ involvement and tumor necrosis was analyzed.

**Results:**

Patient-based SVZ-contact rates in SCM, LGA, and HGA were 24.1%, 68.8%, and 85.4%, respectively. Univariate analysis showed that the SVZ-contact rate was significantly different between SCM and astrocytoma (24.1% vs. 75.2% P < 0.001), also between LGA and HGA (68.1% vs. 85.4% P=0.037). After the tumor volume was adjusted as a covariate, SVZ-contact rates still differed between SCMs and astrocytomas (Odds ratio [OR]: 4.58, 95% Confidence interval [CI]: 1.65 to 12.8, P=0.004). Tumor volume differed between LGA and HGA (P< 0.001), and influenced the association between SVZ involvement and astrocytoma grade (P = 0.05). Among the lateral ventricle regions, the frontal horn was the most frequently involved by astrocytomas. SVZ-contact rates were higher in necrosis group compared with non-necrosis groups (83.9% vs. 50.0%, P < 0.001) among astrocytoma patients. Necrosis positively correlated with SVZ involvement in astrocytomas (r_s_ = 0.342, P < 0.001), but did not correlate with SVZ involvement in SCMs (P = 0.193).

**Conclusions:**

Compared to SCMs, solitary cerebral astrocytomas exhibited spatial proximity to the SVZ, which might distinguish the supratentorial astrocytomas from SCMs.

## Introduction

Glioma is the most common primary tumor occurring in the central nervous system (CNS). Astrocytomas, including glioblastoma (GBM), account for approximately 75% of all gliomas [1]. The incidence of brain metastases is expected to be over 10-fold higher than the incidence of glioblastoma [2]. Approximately 50% of brain metastases appear as solitary brain lesions [3]. Thus, astrocytomas and metastases comprise the majority of solitary tumors in the brain. The therapeutic strategies and prognosis in patients with astrocytomas depend on the precise distinction between high-grade astrocytomas (HGAs; WHO grades III and IV) and low-grade astrocytomas (LGAs; WHO grades I and II). Patients with solitary brain metastases require a systematic work-up to determine the site of the primary carcinoma and detect evidence of any distant metastasis before surgical intervention or medical therapy [4]. Solitary cerebral metastases (SCMs) from known extracerebral primary malignancies can be easily differentiated from astrocytomas on magnetic resonance imaging (MRI). However, SCM with unknown primary malignancies are very difficult to distinguish from astrocytomas, especially HGAs, because of their similar features on standard MRI, such as obvious enhancement and peritumor edema. Similarly, when both low-grade and high-grade gliomas have similar extents of enhancement, necrosis and edema on MRI, their accurate diagnosis is a challenge. Advanced MRI techniques, such as perfusion MRI, diffusion tensor imaging, and MR spectroscopy, have improved the non-invasive grading of astrocytomas[5,6], and to distinguish the metastases from the astrocytomas [7,8]. These techniques require additional imaging time and expense, followed by experienced statistical analyses and interpretation. Overlap in values of tumor and peritumor tissue [8,9,10] from these studies implies that the distinction of astrocytoma and SCM appears to be difficult. Therefore, looking for an easy way to identify astrocytoma and SCM on conventional MRI is very necessary.

The theory of brain tumor stem cells (BTSCs) has aroused widespread concern about the origin of astrocytomas, especially, due to the therapeutic implications. Although BTSCs have been identified in both HGAs and LGAs, these cells are rarely found in LGAs as compared to HGAs [11,12,13]. Mounting evidence has indicated that BTSCs and neural stem cells (NSCs) have similar features such as self-renewal, robust proliferation, migration, and pluripotency [14]. As the largest source of NSCs, the subventricular zone (SVZ) has been shown to be associated with astrocytoma biogenesis and patient prognosis [14,15,16]. SVZ is a region that lies immediately beneath the ependymal layer on the lateral wall of the lateral ventricles [17]. It is reported that appropriate irradiation of the ipsilateral SVZ associated with a significantly improved survival in patients with GBM after gross total resection [18]. However, metastatic emboli tend to occur in areas of sudden reduction in vascular caliber and distal to the vascular field, leading to a metastatic predilection in the vascular border zones and the junction of the gray and white matter [18].

We hypothesized that the space occupying lesions contacting the lateral ventricles on conventional MRI, indicating the SVZ involvement [16,19,20] probably can be used to differentiate SCMs from astrocytomas in the brain. To better characterize the relationship between solitary cerebral tumors and the SVZ, we retrospectively analyzed MRI scans of patients with astrocytomas and SCMs to determine the value of the anatomical relationship between the tumor and the walls of the lateral ventricle, which classified as SVZ contact and SVZ segregate for discriminating astrocytomas from SCMs.

## Methods

### Study population

This retrospective clinical study complied with institutional guidelines and regulations, and was approved by the ethics committee of the First Affiliated Hospital of Xi’an Jiaotong University. Written informed consent was obtained from all subjects. All patient records were anonymized and de-identified prior to the analysis. We reviewed the brain MRI scans of 165 consecutive patients with astrocytomas or metastases presenting as solitary supratentorial space-occupying lesions between May 2004 and November 2011. All selected patients underwent gadolinium diethylenetriamine penta-acetic acid (Gd-DTPA)–enhanced T1-weighted imaging (T1WI) as well as conventional structural MRI, which included T1WI and T2-weighted imaging (T2WI) and/or fluid-attenuated inversion recovery (FLAIR) T2WI. The results were reviewed by two radiologists, and a consensus was reached on the presence of each sign. The reviewers were blinded to the histological diagnosis and clinical history, including the age and sex of the patients. Gross total or near-total resection of tumors was performed in all patients with cerebral astrocytoma and 7 patients with SCM within 1 month after the MRI examination. The other 22 SCM patients had a history of extra-cranial primary malignancy, and underwent resection of the original carcinoma in our hospital. A histopathological diagnosis was obtained in all patients, according to the 2000 WHO histopathology criteria. None of the patients had undergone any therapy prior to the MRI examination. Patients were excluded in the case of ambiguous histopathological diagnosis, tumors located in the lateral ventricle, and inadequate MR imaging.

### MRI acquisition and analysis

All imaging examinations were performed on a 1.5-T MRI (Gyroscan, Release 9.1.2, Philips Medical Systems, The Netherlands) with an eight-channel head coil. MRI sequences included axial fast spin echo T2WI (time of repetition [TR], 4851 ms; time of echo [TE], 120 ms) and axial and sagittal spin echo T1WI (TR, 431 ms; TE, 13 ms). The T1WI sequence was performed before and after the intravenous injection of gadodiamide (Omniscan; GE Healthcare Inc., Cleveland, OH; 0.5 mmol/ml, 0.2–0.4 ml/kg). Eighteen patients underwent additional axial FLAIR T2WI (TR, 6000 ms; TE, 120 ms; time of inversion, 2000 ms). The following parameters were used in all examinations: field of view, 230 mm × 230 mm; matrix, 512 × 512; slice thickness, 5 mm; and inter-slice gap, 1 mm.

The spatial relationship between the tumor and the SVZ was classified as “involvement” or “segregation” by two experienced radiologists. The criteria for SVZ involvement were as follows: contact between any portion of the contrast-enhanced tumor and the wall of the lateral ventricle, or an enhanced line structure between the lateral ventricle wall and the enhanced tumor [16,19]. Necrosis and solid contrast-enhanced tumors were indicated on transverse and sagittal Gd-DTPA—enhanced T1WI. In tumors with necrotic components, the tumor boundary was identified as the enhanced rim or the solid portion. In the case of non-enhanced astrocytomas on Gd-DTPA—enhanced T1WI, the spatial relationship between the tumor and SVZ was evaluated on T2WI or FLAIR T2WI. SVZ involvement was defined as a contact between the edge of the abnormal hyperintense mass and the wall of the lateral ventricle [21,22]. The number of patients with SVZ involvement in a group was divided by the total number of patients in that group to give the patient-based SVZ-contact rate.

The right and left walls of the lateral ventricle were divided into the following four regions (giving a total of eight regions) along the rostrocaudal axis: frontal horn, body, atrium/occipital horn, and temporal horn, as previously described [23]. A relatively large lesion could be in contact with multiple SVZ regions. The frequency of the involvement of each SVZ region in the astrocytoma was determined, and the frequencies of the four regions (data for the left and right sides were combined) were compared statistically.

Tumor volume was measured on the Gd-DTPA—enhanced T1WIs for enhanced lesion and measured on the T2WIs for non-enhanced lesion. Two experienced radiologists independently implemented the measurements of tumor volume (i.e. voxel numbers in tumor region) by using an automated histogram-based thresholding algorithm facilitated by Image J 1.44p software (National Institutes of Health, USA, http://rsb.info.nih.gov/ij/docs/index.html). The actual tumor volume was calculated by the measured voxel numbers × voxel size. The average of the results from the two radiologists was regarded as each patient’s tumor volume.

### Statistical analysis

To evaluate the reliability of the region-based frequency of SVZ involvement in MRI images, we determined the intra- and inter-observer agreements by using kappa statistics. The agreement level was assessed using the criteria reported by Landis and Koch [24]. One-way analysis of variance (ANOVA) was used to test differences in age across the LGA, HGA, and SCM groups. The chi-square test was used to compare between-group differences in the following variables: sex; patient-based SVZ-involvement rate; and region-based frequency of SVZ involvement by astrocytomas. The Fisher exact test was used when the cell count was too small (n < 5).

Spearman correlation coefficients were used to determine the correlation between SVZ involvement and necrosis in MR images of astrocytoma and SCM. The Kruskal—Wallis test was used to compare tumor volume in MR images across the three groups, due to the non-normal data distribution. Logistic regression was used to determine whether tumor volume affected the association between SVZ involvement and astrocytoma grade. The astrocytoma grade was the dependent variable; SVZ contact, tumor volume, and the interaction between them were as independent variables. All data were analyzed using SPSS for Windows, version 13.0 (SPSS Inc., USA). *P* < 0.05 was considered statistically significant.

## Results

### Patient population

**Fig 1:**
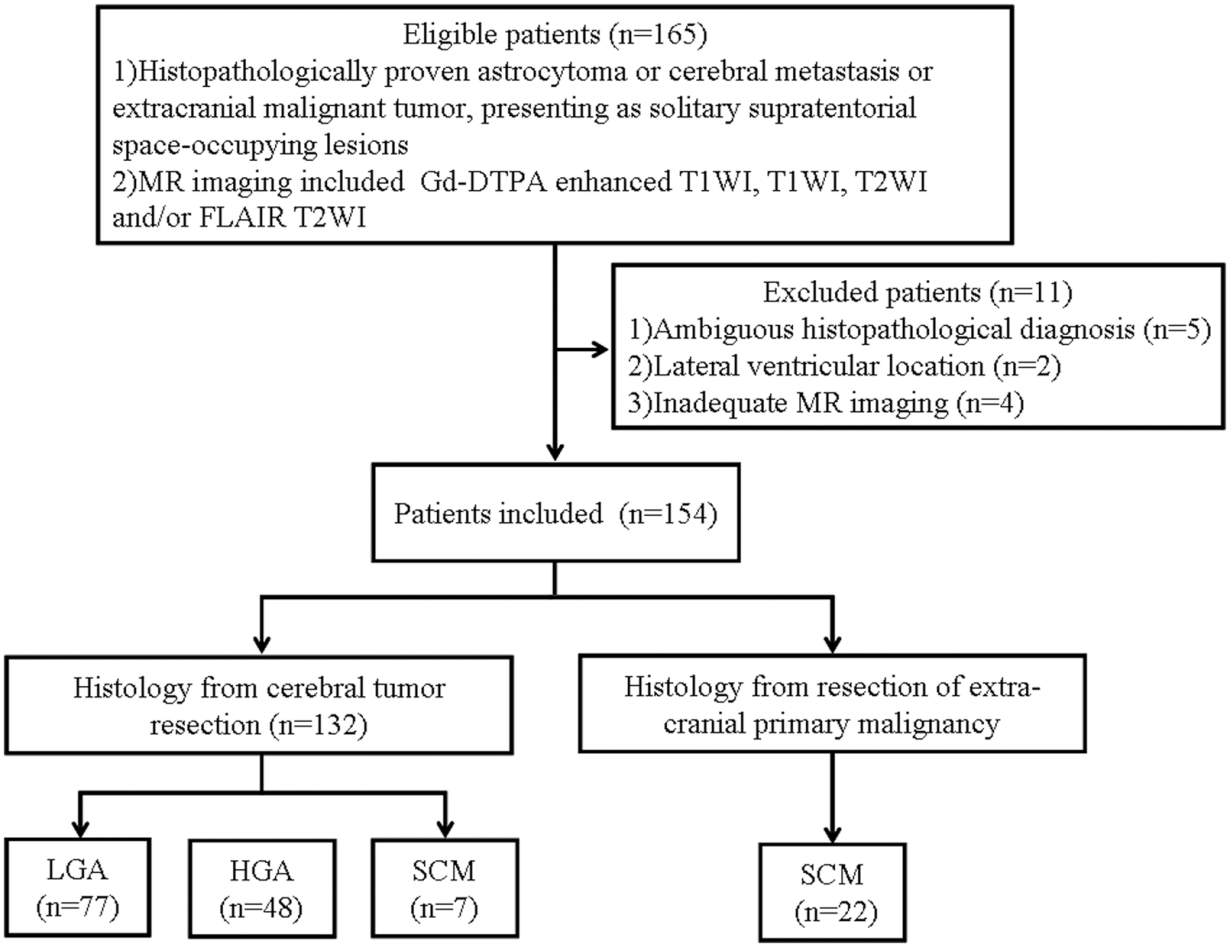
Flow diagram of patient selection and the inclusion and exclusion criteria.

We excluded 11 astrocytoma patients from the study due to the following reasons: (a) ambiguous histopathological diagnosis (n = 5), (b) tumor location in the lateral ventricle (n = 2), and (c) inadequate MRI data due to motion artifacts (n = 3) or susceptibility artifact (n = 1). Thus, finally, 154 patients, including 77 LGA patients, 48 HGA patients, and 29 SCM patients, were enrolled in this study (Fig 1). Among the 154 enrolled patients, there were 91 (59.1%) male and 63 (40.9%) female patients, and their ages ranged from 11 to 76 years, with a mean age of 47.5 ± 14.0 years. There were no significant differences in age and sex across the three groups (Table 1). The primary carcinomas in the 29 SCM patients were located in the lung (n = 11), kidney (n = 7), breast (n = 4), liver (n = 4), thyroid (n = 1), ovary (n = 1), and vagina (n = 1).

**Table 1:** Patient characteristics. LGA, low-grade astrocytoma; HGA, high-grade astrocytoma; SCM, solitary cerebral metastasis.

Characteristic	Study Group			P Value ^§^			
	LGA (N = 77)	HGA (N = 48)	SCM (N = 29)	Overall	LGA vs. HGA	LGA vs. SCM	HGA vs. SCM
**Age, years** *	45.4 ± 14.8	49.3 ± 13.9	50 ± 10.8	0.141	0.116	0.100	0.765
**Male, n (%)**	41 (53.2%)	32 (66.7%)	18 (62.1%)	0.272	0.139	0.415	0.682
**SVZ involvement, n (%)**	53 (68.8%)	41 (85.4%)	7 (24.1%)	<0.001	0.037	<0.001	<0.001
**Tumor volume (ml)** *	25.3 ± 24.2	43.0 ± 25.7	6.1 ± 8.8	<0.001	<0.001	<0.001	<0.001

*Age and tumor volume are expressed as mean ± standard deviation; male sex and SVZ involvement are expressed as frequencies (percentages).

^§^ One-way ANOVA was used to compare the differences in age across the three groups and for pairwise comparisons; the chi-square test was used to compare the overall and between-group differences in gender and SVZ involvement; the non-parametric Kruskal—Wallis test was used to compare the overall and between-group differences in tumor volume.

### Spatial relationship between the tumor and SVZ

**Fig 2:**
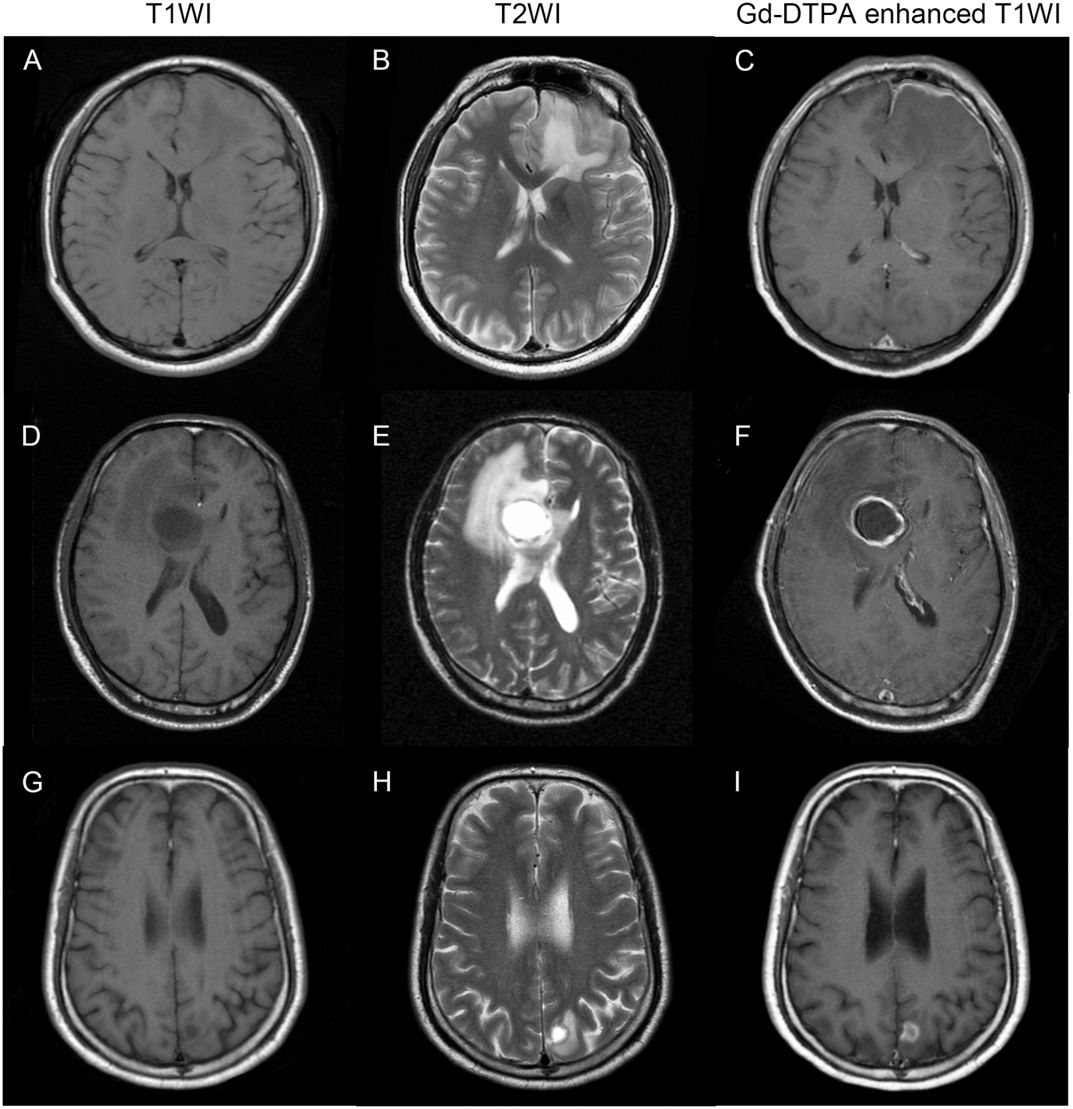
Representative MR images of astrocytoma and solitary cerebral metastasis. A–C: A 39-year-old man with WHO grade I pilocytic astrocytoma without enhancement that is located in the left frontal lobe and in contact with the frontal horn of the left lateral ventricle. D–F: A 47-year-old man with WHO grade III anaplastic astrocytoma that is located in the right frontal lobe, is in contact with the frontal horn of the right lateral ventricle, and exhibits apparent edema and necrosis. G–I: A 50-year-old man with left occipital renal carcinoma metastasis that is not in contact with the lateral ventricle.

Fig 2 shows representative MR images of an LGA, HGA, and SCM, respectively. The patient-based SVZ-contact rates significantly differed across the three groups (overall P < 0.001), between the LGA and SCM groups (68.8% vs. 24.1%, P < 0.001), between the HGA and SCM groups (85.4% vs. 24.1%, P < 0.001), and between the LGA and HGA groups (68.8% vs. 85.4%, P = 0.037; Table 1). In three astrocytoma patients, SVZ involvement was indicated by an enhanced line structure (grade II, n = 1; grade III, n = 2).

Multiple regions of the SVZ were in contact with the astrocytomas in 24 patients, including 12 (15.6%) patients with LGAs and 12 (25%) patients with HGAs. The total number of SVZ regions involved by the astrocytomas was 123. Among the four SVZ regions, the frontal horn was the most commonly involved (n = 45, 36.6%) followed by the occipital horn (n = 35, 28.4%), the temporal horn (n = 23, 18.7%), and the body (n = 20, 16.3%). The frequency of SVZ involvement significantly differed between the frontal horn and the body (P < 0.001), the frontal horn and the temporal horn (P = 0.002), and the body and the atrium/occipital horn (P = 0.022).

### Relationship between tumor size and SVZ involvement

The means and the standard deviations of the tumor volumes in the three groups have been presented in Table 1. Pairwise comparisons showed significant differences in tumor volume between the LGA and SCM groups (25.3 ± 24.2 ml vs. 6.1 ± 8.8 ml, P < 0.001), the HGA and SCM groups (43.0 ± 25.7 ml vs. 6.1 ± 8.8 ml, P < 0.001), and the LGA and HGA groups (25.3 ± 24.2 ml vs. 43.0 ± 25.7 ml, P <0.001; Table 1).

In addition, the univariate analysis showed that astrocytoma patients had larger tumor volume (32.5±26.2 vs. 6.1±8.8, P<0.001) and higher SVZ contacting rates (75.2% vs.24.1%, P<0.001) compared with SCM. When both SVZ involvement and tumor volume were included into a logistic regression model, the two factors turned out to be independently associated with astrocytoma (SVZ involvement OR: 4.58, 95% CI: 1.65 to 12.8, P = 0.004; Volume OR: 1.08, 95% CI: 1.03 to 1.14, P = 0.002), which indicates that the tumor volume did not affect the association between SVZ involvement and the astrocytoma. The interaction of tumor volume and SVZ involvement was also tested in the various tumor types, but was not found to be significant (P = 0.372).

Within astrocytoma patients, univariate analysis showed that HGA group had higher SVZ-contacting rates (P = 0.037) and larger tumor volume (P<0.001) compared with those in LGA group. The P value of the interaction between volume and SVZ involvement was 0.05, indicating that the tumor volume might influence the spatial association between SVZ involvement and astrocytoma grade. We could not perform the further subgroup analyses to explore how the volume affected the association between SVZ and astrocytoma grade due to the limited sample size.

Table 2 shows the levels of agreement (κ) between the two observers for the overall assessment of the spatial relationship between the tumor and SVZ.

**Table 2:** Intra-observer and inter-observer agreements for the assessment of SVZ involvement on MRI.

Category			Consistent regions between two observers				
		Kappa value	Remote	Frontal horn	Temporal horn	Body	Occipital horn
**Intra-observer**	A1-A2	0.851	55	42	17	11	38
	B1-B2	0.923	50	47	22	20	35
**Inter-observer**	A1-B1	0.773	51	47	12	11	32
	A1-B2	0.761	50	45	12	11	33
	A2-B1	0.789	51	41	14	16	32
	A2-B2	0.763	49	39	14	16	32

### Relationship between SVZ involvement and tumor necrosis

In this study, the necrotic regions in astrocytoma were observed in 74.4% (93/125) patients, of which 83.9% (78/93) showed SVZ involvement. Among the remaining 25.6% (32/125) patients with non-necrotic astrocytomas, only 50.0% (16/32) patients showed SVZ involvement. The chi-square test demonstrated a significant difference in the patient-based SVZ-contact rates between the necrosis and non-necrosis groups (P < 0.001). Spearman correlation analysis showed a weak, positive relationship (r_s_ = 0.342, P < 0.001) between SVZ contact and necrosis in the astrocytoma group (Table 3).

**Table 3:** Relationship of SVZ-contact rate with necrosis in astrocytomas and solitary cerebral metastases. SCM, solitary cerebral metastasis.

Category	Astrocytoma		SCM	
	Necrosis	Non-necrosis	Necrosis	Non-necrosis
**Contact with SVZ**	78 (83.9%)	16 (50.0%)	3 (15.8%)	4 (40.0%)
**No contact with SVZ**	15 (26.1%)	16 (50.0%)	16 (84.2%)	6 (60.0%)
**Total**	93	32	19	10
**P-value** *	P < 0.001		P = 0.193	
**Spearman correlation**	r_s_ = 0.342, P < 0.001		r_s_ = -0.269, P = 0.158	

* P-value for comparing the relationship of necrosis and SVZ contact in astrocytomas was calculated using the Pearson chi-square test; for comparing the relationship of necrosis and SVZ contact in SCMs was calculated using the Fisher exact test.

In the SCM, 65.5% (19/29) patients had necrotic tumor components, of which only 15.8% (3/19) showed SVZ involvement in MRI. Among the remaining 10 patients with non-necrotic SCMs, 40% (4/10) showed SVZ involvement in MRI. There was no significant difference in the SVZ-contact rate between the necrosis and non-necrosis groups in SCM patients (P = 0.193). Necrosis of SCMs was not correlated with SVZ involvement (Spearman correlation, r_s_ = -0.269, P = 0.158; Table 3).

## Discussion

In this retrospective MRI study, we found that the patient-based SVZ-contact rate significantly differed between patients with solitary cerebral astrocytomas including WHO grade I-IV and those with SCMs, after the tumor volume was adjusted as a covariate. Additionally, there was a positive correlation between the percentage of necrotic tumors and SVZ involvement in the astrocytoma group but not in the SCM group. These findings suggested that the SVZ is probably the main oncogenesis region in astrocytoma. This raises the possibility that SVZ involvement in MRI may enable differentiation between astrocytomas and SCMs. To our knowledge, no study has yet analyzed the correlation of tumor involvement of the SVZ in a large population of patients with SCMs and different grades of astrocytomas.

In animal studies, the SVZ has shown increased susceptibility to gliomagenesis [25,26]. A radiogenomics study has revealed that SVZ involvement in GBM was associated with 13 genes that accounted for a decrease in hypoxia signatures, changes in copy number, and an increase in tumor vascularity and invasion [20]. These findings suggested that the SVZ is intimately associated with the occurrence and development of gliomas. The overall SVZ-contact rate for astrocytomas was 75.2% in our study, which is lower than the rates reported by Barami et al. [19] and Marsh et al. [27]. There are several potential explanations for the difference between the above studies and ours. We thought the most important reason was the patient composition differentiation in three studies. In present study, the astrocytoma group included all tumor grades (WHO grades I–IV), whereas only grades II–IV, and grades III and IV were included in the studies by Barami et al. and Marsh et al., respectively. Actually, the SVZ-contact rate of grade I was relatively lower than that of grade II, grade III and grade IV, lowered the total SVZ-contact rate of astrocytoma group. Another reason was the evaluation criteria. Marsh et al. defined the anatomic SVZ as an area extending 5 mm from the lateral ventricle in MRI [27]. If we had used this definition of the SVZ compartment same with Marsh et al., the total patient-based SVZ-contact rate of astrocytomas would be elevated from 68.8% to 74.0% in LGA group and did not change in HGA group and SCM group. The total SVZ contact rate of astrocytoma elevated from 75.2% to 78.4%. The kappa values between two criteria evaluation system was 0.94 which indicates that the results of the two methods were consistent.

Low (22%) patient-based SVZ-contact rates of brain metastases were also reported by Barami et al. [19]. This could be due to two reasons. First, the vascular border zones and the junction of gray and white matter are preferred sites for brain metastasis due to the small diameter of the blood vessels or the sudden reduction in vascular caliber in these areas [28], and only a small portion of these areas overlap with the SVZ. Second, SCMs usually expand more homogeneously, and are anticipated to have a spherical morphology [29]. These properties may make contact between SCMs and the SVZ difficult, unlike the case in astrocytomas, which show an invasive growth pattern.

Among the four regions of the lateral ventricle, the frontal horn was most commonly involved by astrocytomas. This result is consistent with other studies [30,31]. They have reported that the densest gliomas occur in the frontal lobe. Barami et al. found that the body of the lateral ventricle was the most common region in contact with gliomas. Quinones-Hinojosa et al. [32] examined the cytoarchitecture and ultrastructure of human lateral ventricles, and found four layers of the SVZ distributed throughout all regions on both sides of the lateral ventricle. This indicated that gliomas could originate from any of the eight ventricular regions. However, the authors observed that proliferating cells were only present in the SVZ of the anterior horn and the body of the lateral ventricle, and not in the other regions of the SVZ [32]. In animal models, regions of the brain with a high degree of cellular proliferation have been found to be more sensitive to chemical or viral oncogenesis than areas with a low proportion of proliferating cells [25,33,34]. This theory reasonably explains our results and those reported by Barami et al. [17]. The discrepancy between the two studies may be attributable to the enrollment of patients with different grades of gliomas.

Large tumor volume has been shown to correlate with a high tumor grade in glioma patients [21,32]. Compared with small tumors, large tumors are associated with relatively high rates of growth and more aggressive behavior. As stated above, SVZ involvement was associated with a decrease in hypoxia signatures, changes in copy number, and an increase in tumor vascularity and invasion [20]. In our study, the patient-based SVZ-contact rates and tumor volume were higher in HGA patients than in LGA patients, which is consistent with the result of the above study [18,24,32]. After the tumor volume was adjusted as a covariate, we found that the SVZ-contact rate still differed between the astrocytoma and SCM groups, suggesting that SVZ involvement in MRI could differentiate between astrocytomas and SCMs.

The incidence of necrosis was similar in the astrocytoma group (74.4%) and the SCM group (65.5%). Therefore, we further assessed the relationship between necrosis and SVZ involvement by using the chi-square test. The results implied that in astrocytomas, contact with the SVZ correlated with a higher possibility of necrosis (P < 0.001, R = 0.342). Necrosis in glioma is linked to hypoxia and angiogenesis, which are key factors for maintaining the characteristics of BTSCs and for tumor evolution [35]. Recently, Mendez et al. demonstrated that hypoxia-inducible factor-1 alpha (HIF-1α) is involved in the survival and self-renewal of BTSCs [36]. Additionally, Bao et al. indicated that tumors generated from a CD133+ glioma cell population displayed increased tumor vascularity, necrosis, and hemorrhage [37]. Therefore, the microenvironment and cellular composition of the SVZ play a vital role in astrocytoma progression and occurrence of necrosis. In SCMs, necrosis was not shown to correlate with SVZ involvement. Zhong et al. found that the HIF-1α protein was overexpressed in 13 of 19 human cancers with regional and distant metastases, including colon, breast, gastric, lung, skin, ovarian, pancreatic, prostate, and renal carcinomas [38]. Their study suggested that the necrosis in SCMs could be associated with the type of primary carcinoma rather than the location in the brain.

Our study has some limitations. First, the relatively small number of patients with grade IV astrocytomas and the fact that we combined the data for astrocytoma grades I and II, and grades III and IV might have affected the grade-specific patient-based SVZ-contact rates. However, the increase in SVZ-contact rates with increasing WHO grade of gliomas was reasonable in theory, and in agreement with previous reports [19,27,39]. Second, not all SCMs were pathologically verified. Third, it was difficult to accurately delineate the boundary of astrocytomas and the SVZ on MRI. Fourth, although the patient-based SVZ-contact rate differed between LGAs and HGAs in this retrospective study, there was an interaction between tumor volume and SVZ involvement. Thus, a further analysis based on a large sample size is required to clarify the relationship of tumor size with SVZ involvement in patients with astrocytomas of different grades.

In summary, SVZ involvement is potentially related to the origin, position, and necrosis of astrocytomas. In contrast, the position and necrosis of SCMs may be decided by the vascular anatomy and the histopathological type of the primary tumor, and may not be related to SVZ involvement. Thus, compared with SCMs, astrocytomas show more intimate contact with the SVZ. In conclusion, the space occupying lesions contacting the lateral ventricles in MRI, which indicated the SVZ involvement, can be used as a complementary support for distinguishing astrocytomas and SCMs in clinical practice.
